# Supplementary material for: Favipiravir-resistant influenza A virus shows potential for transmission
Source: PLoS Pathog. 2021 Jun 1;17(6):e1008937. doi: 10.1371/journal.ppat.1008937 (PMC8195362; doi:10.1371/journal.ppat.1008937)
Supplement: S1 Appendix — (PDF) [file ppat.1008937.s001.pdf]

Modelling sensitivity analysis for “’Favipiravir-resistant influenza A virus shows potential for transmission’

This sensitivity analysis explores the conditions under which reassortment is necessary to produce the key observation that the proportion of virus carrying the PB1 K229R mutation decreases over time in the donor, but the proportion of virus carrying the PA P653L mutation does not.

The default model parameters are

Parameter	Definition	Value
μ	Mutation rate	2×10^{-4} per generation
f_{MW}	Fitness of PB1 K229R relative to WT	0.01
f_{WM}	Fitness of PA P653L relative to WT	1.25
f_{MM}	Fitness of double mutant relative to WT	1
MOI	Multiplicity of infection	1
V	Viral load	10^6 virions
b	Mean burst size for a cell infected by one WT virion	10 virions
p_{MW}	Initial proportion of virus with PB1 K229R only	0
p_{WM}	Initial proportion of virus with PA P653L only	0
p_{MM}	Initial proportion of double mutant virus	0.95

The mutation rate was obtained from Pauly et al. [2017]. The relative fitnesses f_{MW}, f_{WM} and f_{MM} are chosen to qualitatively reflect the result in Goldhill et al. [2018] that in MDCK cells, the fitness of the double mutant is comparable to the wild type, the PA P653L single mutant has a slight fitness advantage, and the fitness of the PB1 K229R single mutant is much less than wild type. We will see below that the result that reassortment and the fitness advantage of the PA P653L single mutant drive a decrease in the observed frequency of the PB1 K229R segment does not depend on the precise value of the fitness of the double mutant, or the PB1 K229R single mutant, provided that it does not have a fitness advantage. However, it is sensitive to the precise fitness advantage of the PA P653L single mutant. The initial proportion of each strain, p_{MW}, p_{WM} and p_{MM} were chosen to reflect the inoculum in the ferret experiments. The default MOI of 1 was chosen based on data by Fukuyama et al. [2015], which showed that in mice infected with four influenza strains of equal fitness, at day 2 post-infection, the ratio of cells infected with multiple

strains to cells infected with a single strain is approximately 0.4. An MOI of 1 would predict a ratio of 0.51 in this situation, which is broadly consistent with the experimental observation. The viral load in virions is chosen based on the viral load in nasal wash between days 2 and 5 of our experimental data, which is approximately 10^5 pfu/mL. The concentration of virions in nasal wash is lower than the concentration of virions in the respiratory tract by a factor of 1-100 [Handel et al., 2007]. The volume of the ferret respiratory tract is about 1 mL [Petrie et al., 2013]. Therefore the number of pfu in the ferret respiratory tract is 10^5-10^7 pfu. We assume that one pfu equals one infectious virion, and choose 10^6 pfu as the geometric mean over the plausible range.

First, we consider changes to the baseline fitness parameters. Figure 1 shows that if the fitness advantage of the PA P653L single mutant increased to 1.5 or above, the frequency of the PB1 K229R segment would decrease over time even without reassortment. Figure 2 shows that if the double mutant had a fitness advantage/cost relative to WT, and reassortment were absent, then single mutants would be largely absent, which cannot explain the decoupling of the proportion of mutants for each segment. Under this scenario, reassortment is required to explain the decrease in the PB1 K229R segment without a decrease in the PA P653L segment. Figure 3 shows that regardless of the relative fitness of the PB1 K229R single mutant, as long as it is equal to or below 1, then reassortment is required to observe a large increase in the PA P653L single mutant, leading to a large decrease in the proportion of the PB1 K229R segment.

Then, we consider changes in the inoculum composition. Figure 4 shows that if the inoculum consists of wild type and PB1 K229R + PA P653L double mutant only, then regardless of the proportion of wild type virus in the inoculum, reassortment is required to observe a large increase in the PA P653L single mutant, leading to a large decrease in the proportion of the PB1 K229R segment. Without reassortment, increasing the initial proportion of wild type virus only decreases the proportion of PB1 K229R + PA P653L double mutant, and increases the proportion of wild type virus at 20 generations; then proportions of both the PB1 K229R segment and the PA P653L segment would decrease over time, which was not observed. Figure 5 shows that if the initial proportions of wild type, PB1 K229R single mutant and PA P653L single mutant are equal, with the PB1 K229R + PA P653L double mutant making up the remainder, then reassortment is not required to observe a large increase in the PA P653L single mutant and thus a large decrease in the PB1 K229R segment. This is because reassortment is only required to quickly generate the PA P653L single mutant, after which its fitness advantage alone is sufficient for it to grow in proportion; this is not necessary if the PA P653L single mutant is already present in the inoculum.

Then, we consider changes to the mutation rate per generation, the MOI and the viral load. Figure 6 shows that if the mutation rate were increased tenfold to 2×10^{-3}, a decrease in the PB1 K229R segment would be observed without reassortment. Figure 7 shows that regardless of the MOI, mutation alone is insufficient to reproduce a large increase in the PA P653L single mutant. When there is reassortment, the proportion of the PA P653L single mutant increases

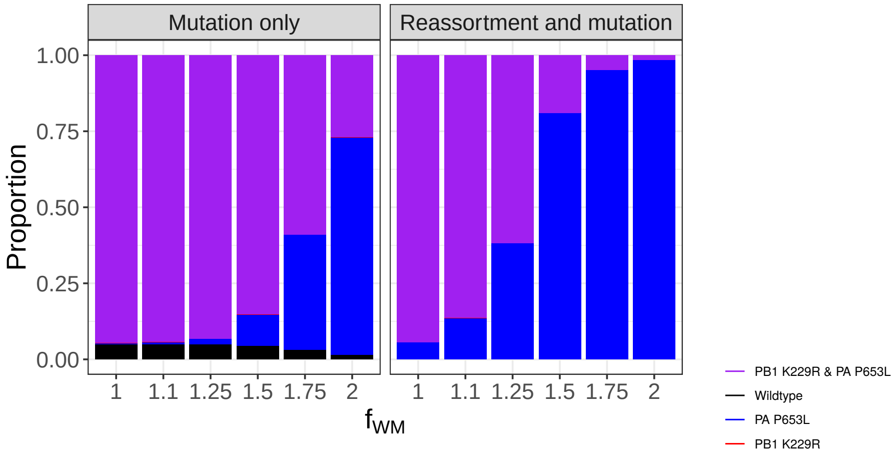

Figure 1: Proportion of each strain for the indicated relative fitness for the PA P653L single mutant, for (left) mutation only and (right) mutation and reassortment, at the 20th generation. The default value is $f_{WM} = 1.25$.

for MOIs of 0.1 and 1, but this effect is smaller for an MOI of 10. This is because when a cell is infected with the PA P653L single mutant only, then the burst size is larger and most progeny are the PA P653 single mutant (except for output virions where mutation has occurred); however, when a cell is co-infected with the PA P653L single mutant and a different virus, although the burst size is still larger, we have assumed that the composition of the progeny is evenly split between the infecting strains. Therefore, the co-infecting strains also enjoy the fitness advantage of the P653L single mutant. An intermediate MOI ensures that enough co-infection occurs for the P653L single mutant to be generated in the first place, while still having enough singly infected cells for its fitness advantage to manifest. Figure 8 shows that results do not vary with the viral load.

Last, we consider changes to the model structure. Figure 9 shows the proportion of each strain assuming that the burst size from an infected cell is independent of the number of infecting virions, rather than increasing linearly with it; and assuming that the proportion of mutants for each segment produced by an infected cell depends on the mutation's fitness rather than only the proportion of mutant virions infecting that cell). In both cases, reassortment is required to reproduce our observations that the proportion of virus carrying the PB1 K229R mutation decreases over time in the donor, but the proportion of virus carrying the PA P653L mutation does not.

In summary, unless there are large increases in the fitness advantage of PA P653L and/or the mutation rate relative to the baseline parameters, or the PA P653L single mutant is already present in the inoculum, reassortment is required to explain the observed decrease in the PB1 K229R segment.

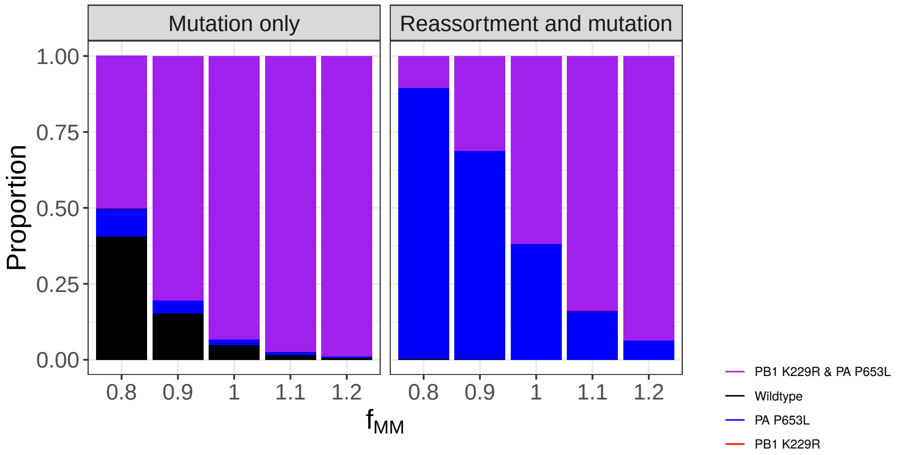

Figure 2: Proportion of each strain for the indicated relative fitness for the PB1 K229R + PA P653L double mutant, for (left) mutation only and (right) mutation and reassortment, at the 20th generation. The default value is $f_{MM} = 1$.

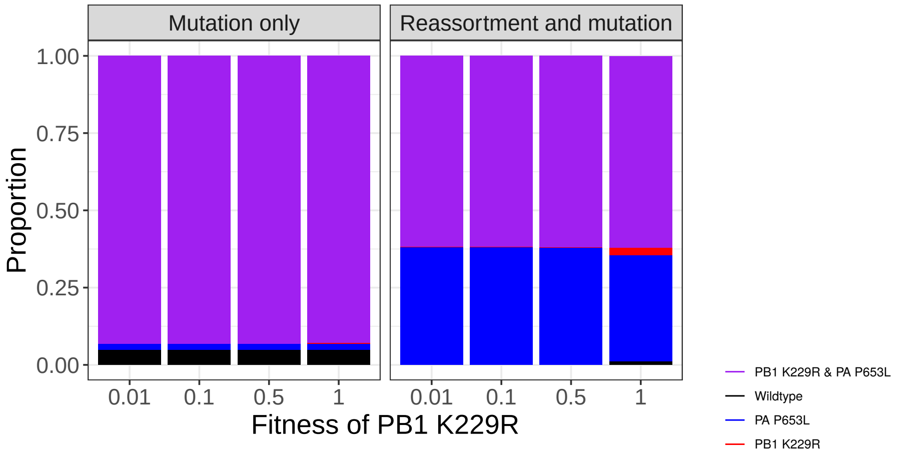

Figure 3: Proportion of each strain for the indicated relative fitness for the PB1 K229R single mutant, for (left) mutation only and (right) mutation and reassortment, at the 20th generation. The default value is $f_{MW} = 0.01$.

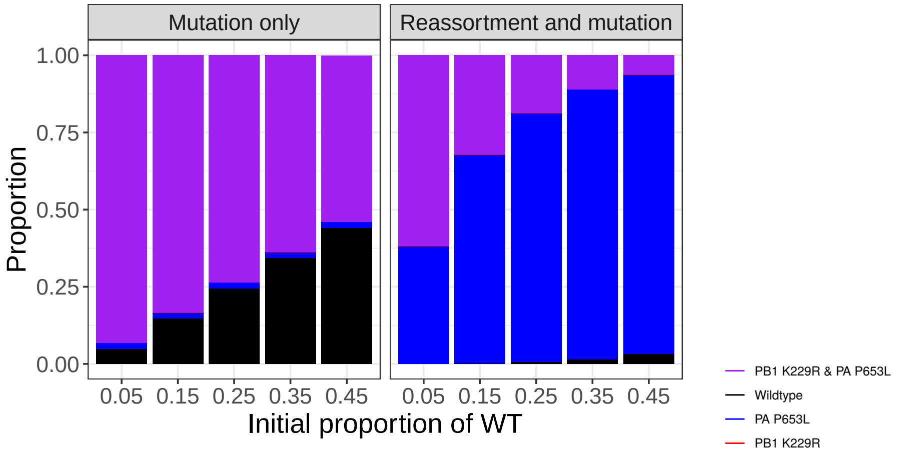

Figure 4: Proportion of each strain for the indicated initial proportion of wild type virus, for (left) mutation only and (right) mutation and reassortment, at the 20th generation. The default value is 0.05. We assume that the rest of the inoculum is the PB1 K229R + PA P653L double mutant, i.e. there are no single mutants in the inoculum.

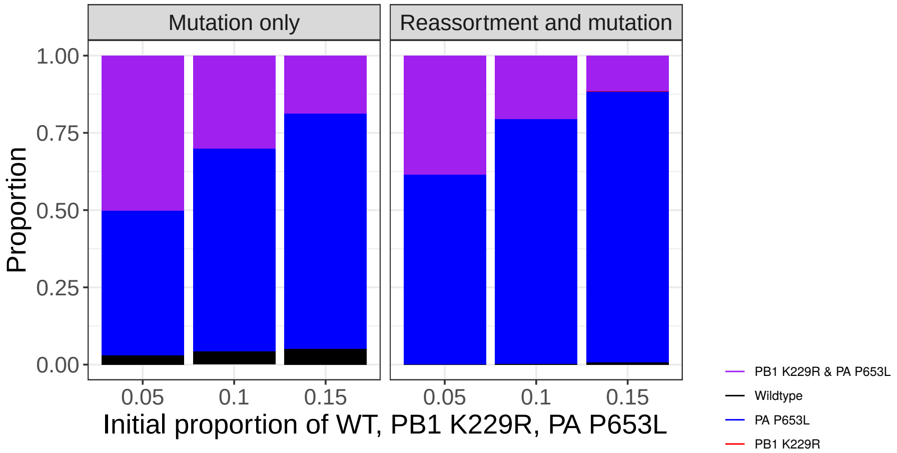

Figure 5: Proportion of each strain for the indicated initial proportions of wild type, PB1 K229R single mutant, and PA P653L single mutant, for (left) mutation only and (right) mutation and reassortment, at the 20th generation. For example, the 0.05 bar is when the inoculum is 5% wild type, 5% PB1 K229R single mutant, 5% PA P653L single mutant, and 85% PB1 K229R + PA P653L double mutant.

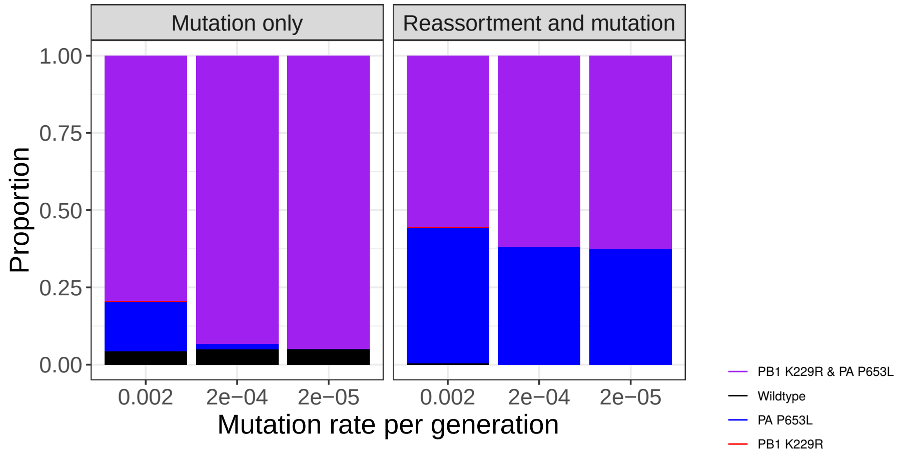

Figure 6: Proportion of each strain for the indicated mutation rates, for (left) mutation only and (right) mutation and reassortment, at the 20th generation. The default value is $\mu = 2 \times 10^{-4}$ per generation.

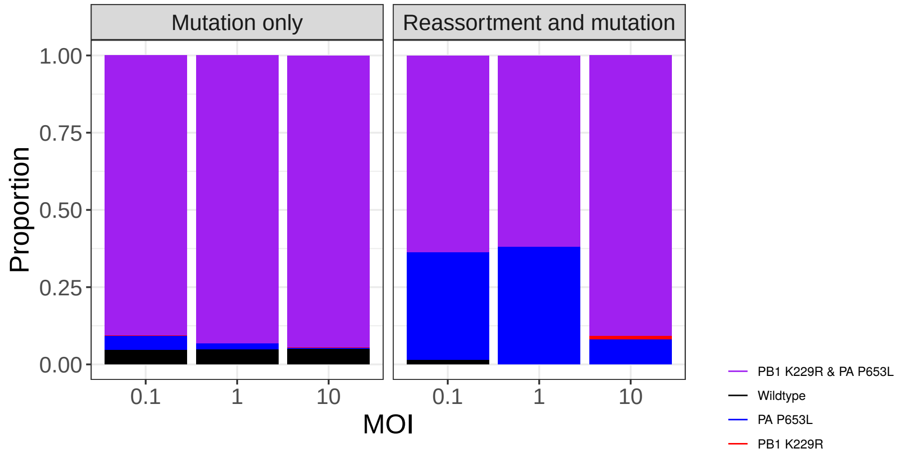

Figure 7: Proportion of each strain for the indicated MOI, for (left) mutation only and (right) mutation and reassortment, at the 20th generation. The default value is MOI = 1.

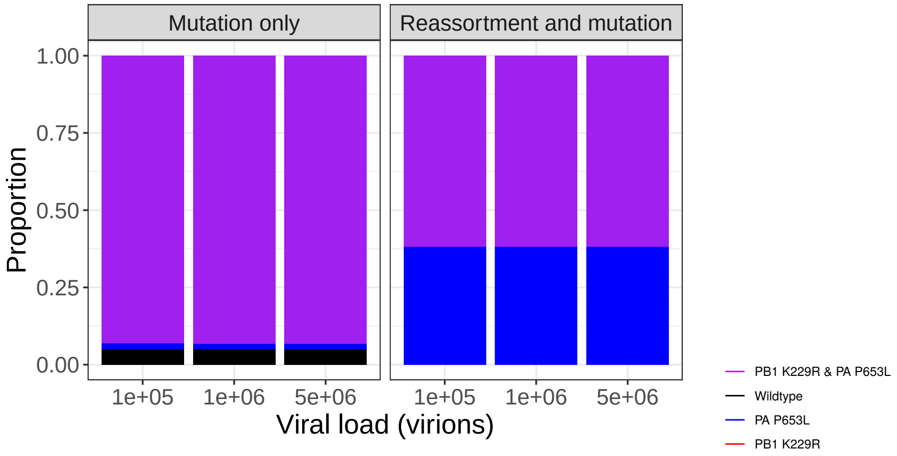

Figure 8: Proportion of each strain for the indicated viral load, for (left) mutation only and (right) mutation and reassortment, at the 20th generation. The default value is 10^6 virions.

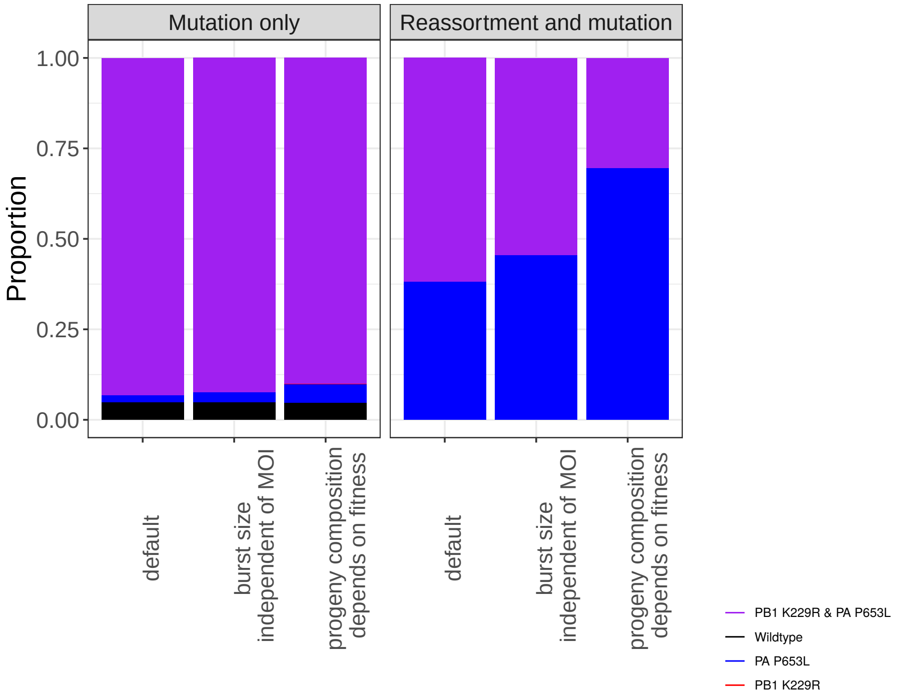

Figure 9: Proportion of each strain for the indicated model changes, for (left) mutation only and (right) mutation and reassortment, at the 20th generation.

References

S. Fukuyama, H. Katsura, D. Zhao, M. Ozawa, T. Ando, J. E. Shoemaker, I. Ishikawa, S. Yamada, G. Neumann, S. Watanabe, H. Kitano, and Y. Kawaoka. Multi-spectral fluorescent reporter influenza viruses (Color-flu) as powerful tools for in vivo studies. *Nature Communications*, 6(1): 6600, Mar. 2015. ISSN 2041-1723. doi: 10.1038/ncomms7600. URL `https://www.nature.com/articles/ncomms7600`. Number: 1 Publisher: Nature Publishing Group.

D. H. Goldhill, A. J. W. te Velthuis, R. A. Fletcher, P. Langat, M. Zambon, A. Lackenby, and W. S. Barclay. The mechanism of resistance to favipiravir in influenza. *Proceedings of the National Academy of Sciences*, 115(45):11613–11618, 2018. ISSN 0027-8424. doi: 10.1073/pnas.1811345115. URL `https://www.pnas.org/content/115/45/11613`.

A. Handel, I. M. L. Jr, and R. Antia. Neuraminidase Inhibitor Resistance in Influenza: Assessing the Danger of Its Generation and Spread. *PLOS Computational Biology*, 3(12):e240, Dec. 2007. ISSN 1553-7358. doi: 10.1371/journal.pcbi.0030240. URL `https://journals.plos.org/ploscompbiol/article?id=10.1371/journal.pcbi.0030240`. Publisher: Public Library of Science.

M. D. Pauly, M. C. Procario, and A. S. Lauring. A novel twelve class fluctuation test reveals higher than expected mutation rates for influenza a viruses. *eLife*, 6:e26437, jun 2017. ISSN 2050-084X. doi: 10.7554/eLife.26437. URL `https://doi.org/10.7554/eLife.26437`.

S. M. Petrie, T. Guarnaccia, K. L. Laurie, A. C. Hurt, J. McVernon, and J. M. McCaw. Reducing Uncertainty in Within-Host Parameter Estimates of Influenza Infection by Measuring Both Infectious and Total Viral Load. *PLOS ONE*, 8(5):e64098, May 2013. ISSN 1932-6203. doi: 10.1371/journal.pone.0064098. URL `https://journals.plos.org/plosone/article?id=10.1371/journal.pone.0064098`. Publisher: Public Library of Science.
