# Supplementary figures and images for: Favipiravir-resistant influenza A virus shows potential for transmission
Source: PLoS Pathog. 2021 Jun 1;17(6):e1008937. doi: 10.1371/journal.ppat.1008937 (PMC8195362; doi:10.1371/journal.ppat.1008937)

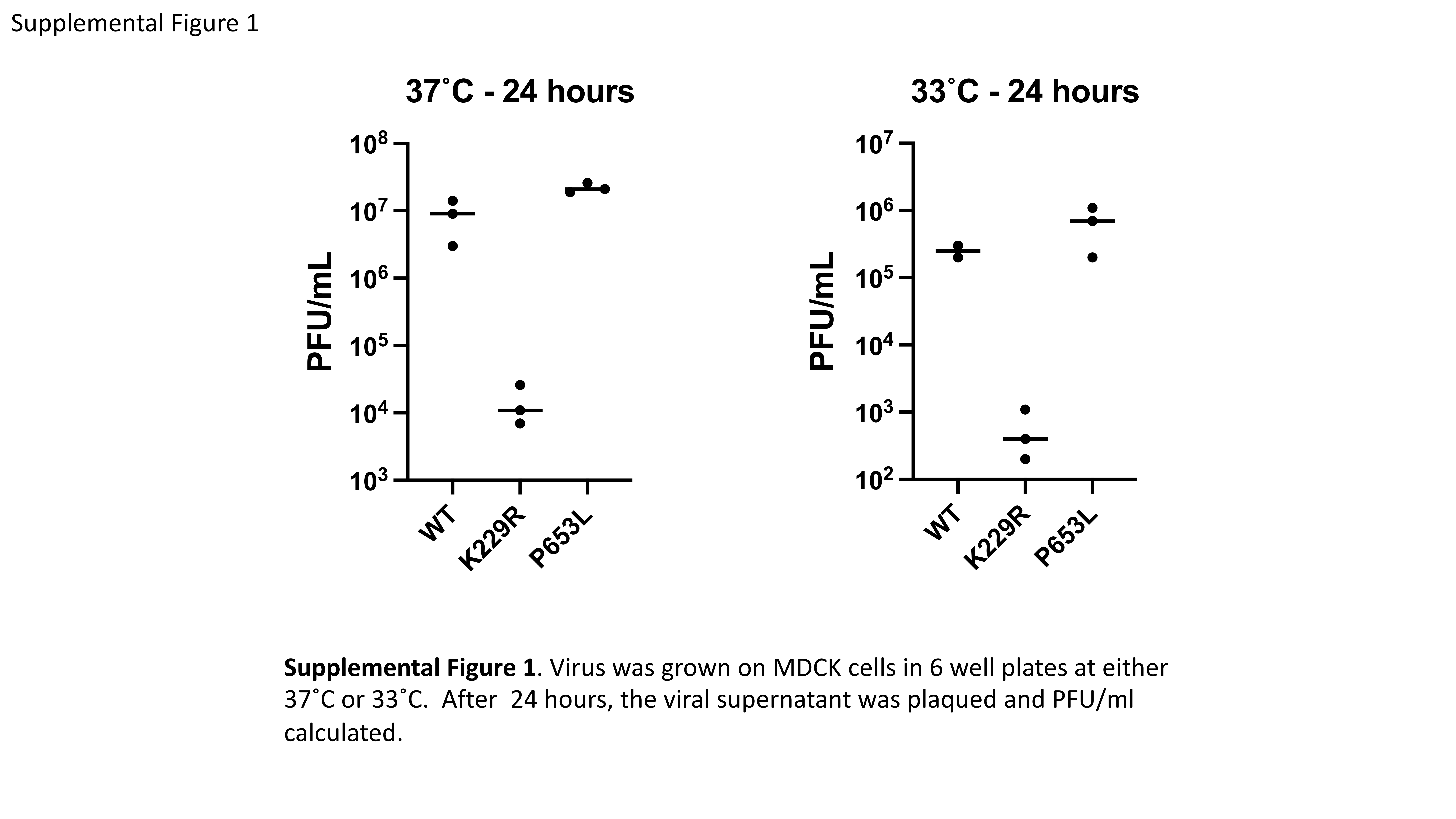

Supplement: S1 Fig — After 24 hours, the viral supernatant was plaqued and PFU/ml calculated. (TIFF) [file ppat.1008937.s002.tiff]

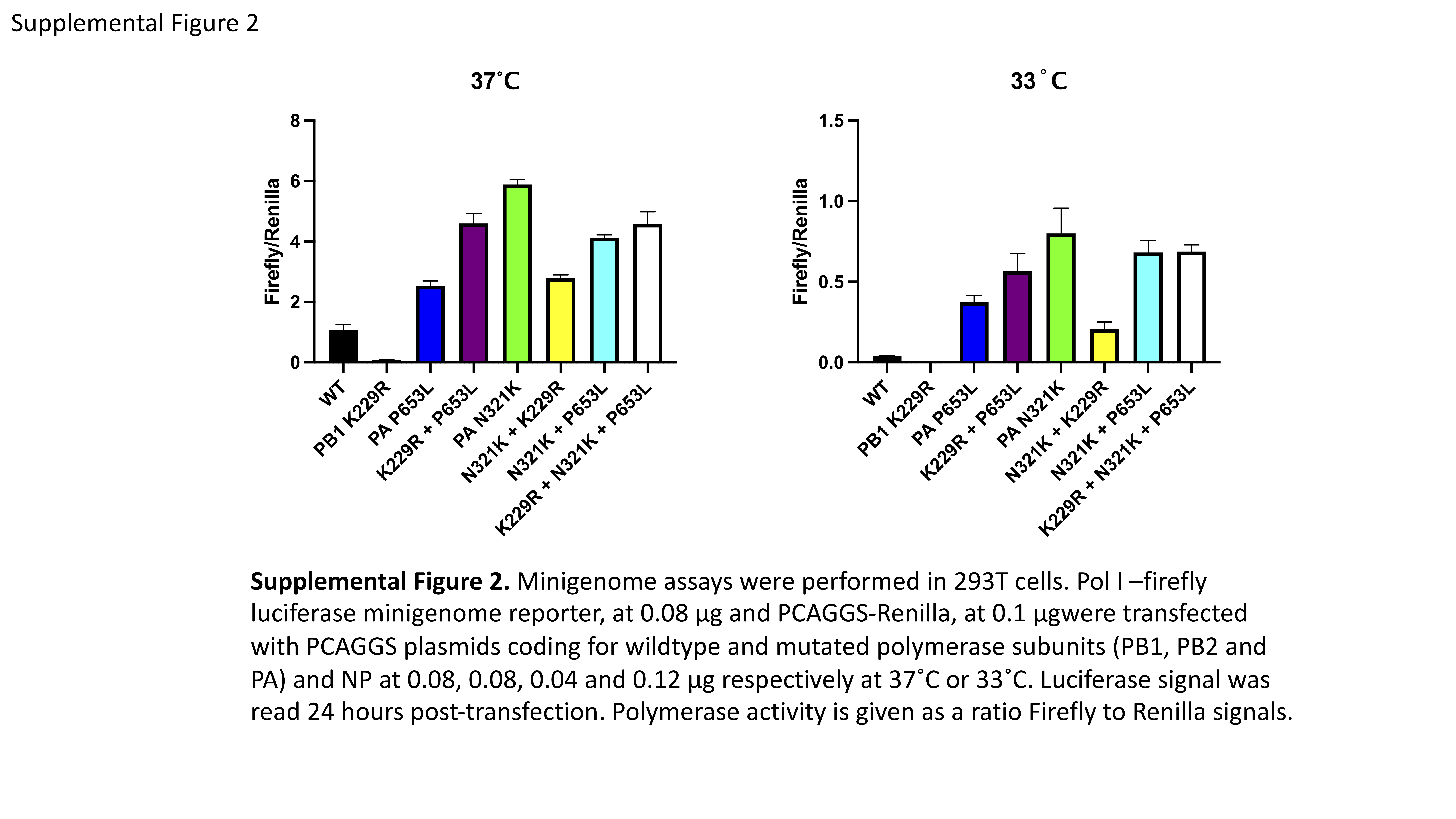

Supplement: S2 Fig — Pol I–firefly luciferase minigenome reporter, at 0.08 μg and PCAGGS-Renilla, at 0.1 μgwere transfected with PCAGGS plasmids coding for wildtype and mutated polymerase subunits (PB1, PB2 and PA) and NP at 0.08, 0.08, 0.04 and 0.12 μg respectively at 37°C or 33°C. Luciferase signal was read 24 hours post-transfection. Polymerase activity is given as a ratio Firefly to Renilla signals. (TIFF) [file ppat.1008937.s003.tiff]

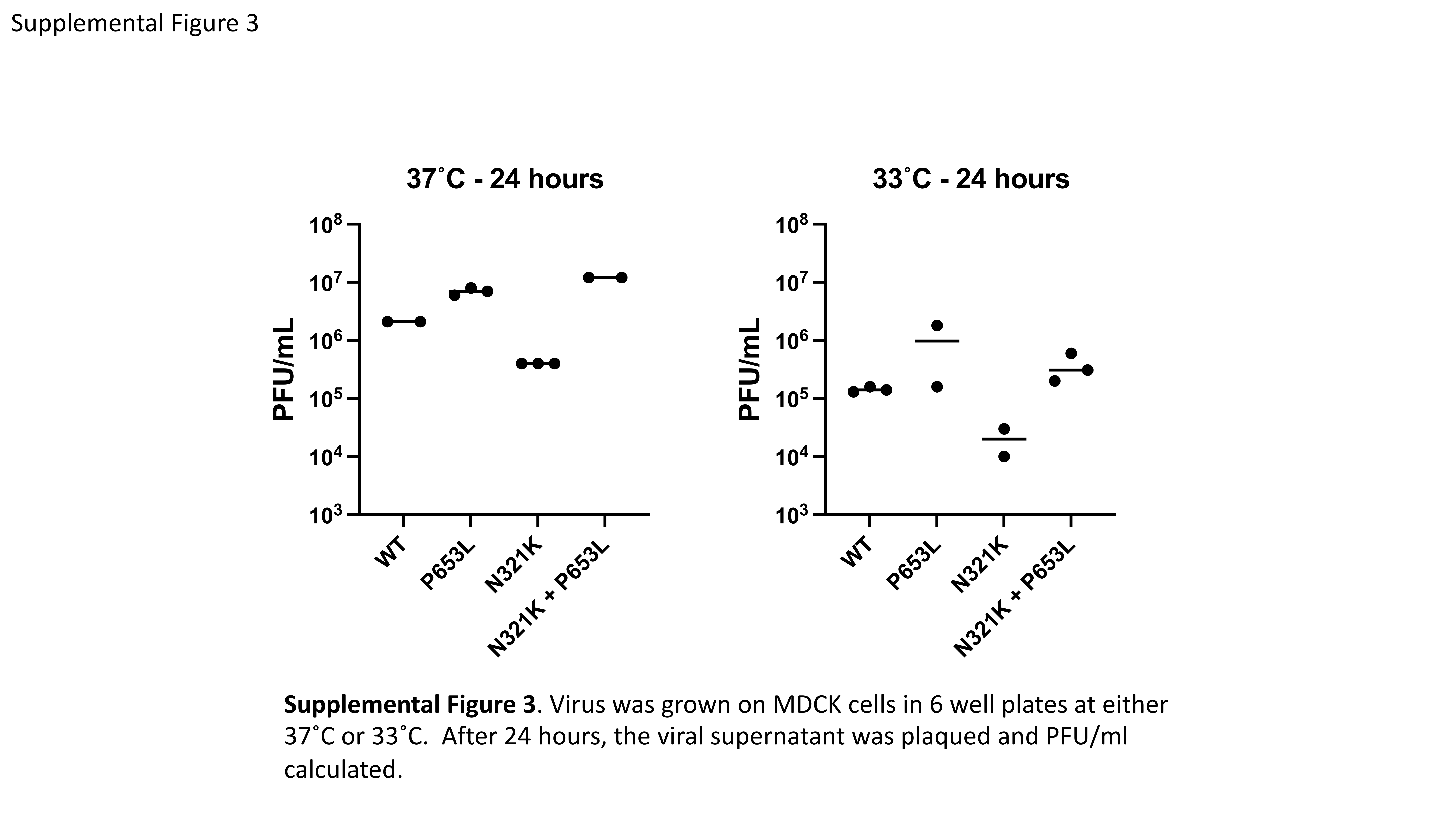

Supplement: S3 Fig — After 24 hours, the viral supernatant was plaqued and PFU/ml calculated. (TIFF) [file ppat.1008937.s004.tiff]

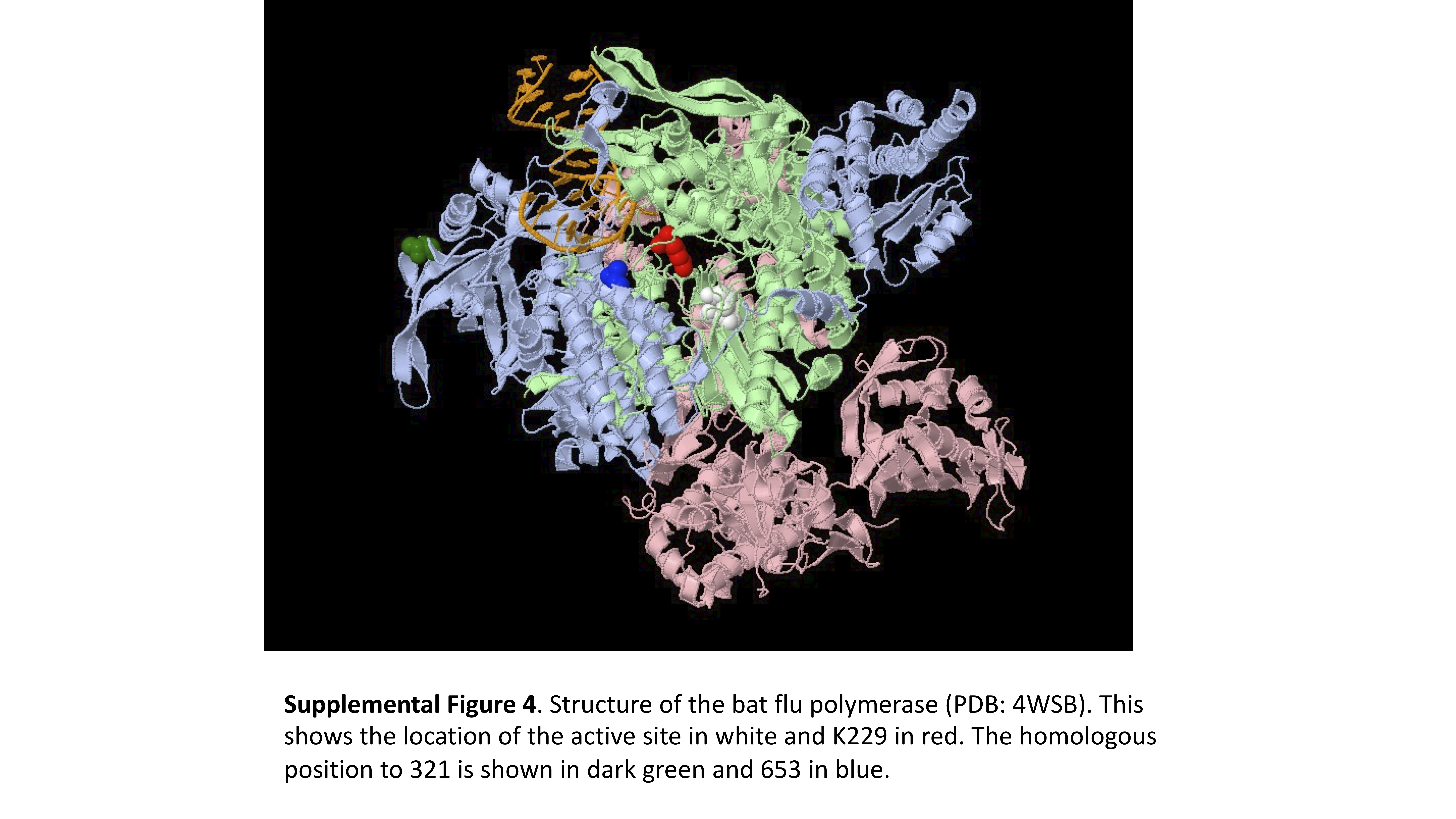

Supplement: S4 Fig — This shows the location of the active site in white and K229 in red. The homologous position to 321 is shown in dark green and 653 in blue. (TIFF) [file ppat.1008937.s005.tiff]
