# Supplementary material for: Favipiravir-resistant influenza A virus shows potential for transmission
Source: PLoS Pathog. 2021 Jun 1;17(6):e1008937. doi: 10.1371/journal.ppat.1008937 (PMC8195362; doi:10.1371/journal.ppat.1008937)
Supplement: Attachment — Submitted filename: Reviewers Comments.docx [file ppat.1008937.s006.docx]

**Part I - Summary**
Please use this section to discuss strengths/weaknesses of study, novelty/significance, general execution and scholarship.

Reviewer #1: Goldhill et al., performed transmission studies with a favipiravir-resistant strain of 2009 pandemic H1N1. The resistant virus was previously generated on the A/England/195/2009 (H1N1) background and contains the PB1 K229R and PA P653L mutations. Herein the authors evaluated the in vivo fitness effects of these resistance mutations. When the A/England virus carrying the PB1 K229R+ PA P653L mutations was inoculated into ferrets at a ratio of 95% mutant virus to 5% wild-type, the resistant virus was capable of both direct contact and airborne transmission. However, reassortant viruses carrying the PA P653L mutation with the wild-type PB1 also transmitted and eventually became the dominant strain. Subsequently, polymerase assays were performed to determine the fitness effect of the PA 653L mutation. These studies show that the PA mutation enhances polymerase activity in the A/England/195/2009 (H1N1) strain. When this mutation was introduced into a more recent pandemic H1N1 isolate, the PA P653L mutation did not confer enhanced polymerase activity. This was due to an additional mutation, N321K, that enhances polymerase activity in more recent pandemic H1N1 strains.

Reviewer #2: Goldhill et al. use an elegant combination of approaches to examine the within- and between-host dynamics of an IAV favipiravir escape mutant during mixed infection with wild type virus. In particular, a double mutant Eng195 (pH1N1) virus is examined that contains both a PB1 K229R escape mutation and a PA P653L compensatory mutation. Mixed infection with wild type virus is performed and relative proportions of the wild type and mutant segments are monitored by deep sequencing of nasal swabs from inoculated, direct contact and aerosol contact ferrets. The results reveal transmission of the escape mutant virus, but also a decline in its prevalence over time within multiple ferrets. The particular dynamics observed suggest that the PA P653L (compensatory) mutation is uncoupled from PB1 K229R by reassortment, and that this PA mutation undergoes positive selection. This model is tested in a simple but robust computational simulation; the results of which support the model given. Since the PA P653L mutation appears to be driving the dynamics observed but is not seen in circulating pH1N1 viruses, the authors then extend their thinking to recent pH1N1 viruses. Recent strains have higher baseline polymerase activity owing to a PA N321K polymorphism. In the context of PA N321K, or a full Eng687 strain background, PB1 K229R and PA P653L mutations have lesser and no effect, respectively, on pol activity. The authors conclude that the fate of 229R favipiravir resistance mutations will depend in part on the particular strain background and that resistance may arise more easily in recent pH1N1 viruses compared to 2009 pandemic isolates.

This is an interesting and elegant study, which not only gives insight into the potential for favipiravir resistance to spread but also reveals the factors governing within- and between-host dynamics in mixed IAV infection.

Reviewer #3: In this study, the authors build on their previous work, in which they showed in vitro that the favipiravir resistance mutation PB1 K229R, which conferred a fitness cost, could be compensated for by a PA P653L mutation. Here, they use a ferret model to ask whether this compensated resistance phenotype could spread in vivo, and hence whether favipiravir resistance could spread in human influenza viruses.
The paper is well conceived and for the most part well-written (other than the discussion of the model, where I confess I got a bit bogged down, see below). The experimental work is well-performed and the overall aims of the paper have significant importance. However, in my opinion the paper as written has three key weaknesses (at least two of which could be addressed without the need for further experiments).

**Part II – Major Issues: Key Experiments Required for Acceptance**
Please use this section to detail the key new experiments or modifications of existing experiments that should be absolutely required to validate study conclusions.

Generally, there should be no more than 3 such required experiments or major modifications for a "Major Revision" recommendation. If more than 3 experiments are necessary to validate the study conclusions, then you are encouraged to recommend "Reject".

Reviewer #1: 1) The authors utilize viral polymerase activity assays to infer fitness effects of mutations in different viral strains. While these results are valid, they do not directly measure viral fitness, and the standard in the field is to perform viral growth kinetics studies to complement polymerase assays. Thus, additional in vitro growth kinetic studies of the mutant viruses are required. Moreover, the authors do not indicate the temperature at which the polymerase assays were performed. From review of the methods, this is most likely at 37ºC. Richard et al., (Nature Communications, 2020) demonstrated that during transmission between ferrets, viruses from the upper respiratory tract are transmitted. Therefore, both polymerase and growth kinetics studies should be performed at 33ºC.

**Our previous work suggested that there was a fitness advantage in MDCK cells for the viruses containing only the P653L mutation. The P653L mutation also greatly increased the polymerase activity in a minigenome assay. We have now confirmed that this fitness advantage is present in MDCK cells grown at both 33 and 37˚C (Supplementary Figure 1). Figure 4 showed that the N321K mutation in PA was likely the reason why P653L has not evolved. P653L has an advantage in the minigenome assay over wildtype but does not further increase polymerase activity in the presence of N321K. We confirmed that these minigenome results were replicable at 33C (Supplementary Figure 2). We performed viral growth assays in MDCKs comparing the growth of N321K and P653L and found surprisingly that N321K grew worse than wildtype (Supplementary Figure 3). This is contrary to what was seen in minigenome assays as well as HAE cell growth curves from Elderfield et al. 2014. This lack of an advantage to N321K suggests that either that it interacts with other mutations in Eng687 or that the fitness advantage is only realised in more humanlike systems.**

Reviewer #2: none

Reviewer #3: (1) Discussion of mutations. The authors detected nucleotide-level changes but only express them as amino-acid level changes. This makes sense for the modelling sections, but when handling experimental data it means that they ignore key information that would be useful for identifying selection vs contamination or reinfection, an issue that comes up several times in the paper. Specifically, where multiple synonymous variants could give the same aa, the detection of a novel nt sequence encoding the original aa would suggest selection for that aa, in a way that detection of the original nt sequence would not. And a separate point: when describing changes in genotype frequency and detection of mutations, a statement of the depth of coverage would be helpful, as would an assessment of the intrinsic error rate of the NGS used.

**It would have been nice to be able to distinguish between mutations arising from different causes but we did not see this in our sequencing as all samples had the same codons coding for the amino acids. There was no variation at 1^st^ or 3^rd^ base position in our sequencing and therefore it was not possible to determine the origin of mutations by looking at other synonymous variants. The average depth of coverage was 12899 (min 208). It is generally accepted that Illumina NGS can distinguish between mutations differing by 1% (e.g. Pfeiffer et al. Scientific Reports 2018) which was adequate for this study.**

(2) Which virus is interesting? The paper focusses most of its content on discussing work on A/England/195/2009 before concluding that subsequent changes in genotype render the fitness compensation constellation that had been studied irrelevant to more recent pH1N1 viruses (such as A/England/687/2010, and presumably all since). This is an important point, and the authors have my sympathy as it was probably very frustrating to discover. The reader is currently left wondering: ‘now that they’ve found this effect in a virus that is no longer circulating, what might it mean for viruses that are still out there?’ How best to address this is a balance of effort vs impact that should probably be decided in discussion with the editor. I can see that it could be ethically and financially difficult to justify repeating the ferret studies with other viruses. However, it would presumably be entirely feasible to consider the effects of fitness compensation on different backgrounds using a version of the model in Fig 3. It would also be feasible to repeat Fig 4 on a wider range of genetic backgrounds – indeed, the authors have already done much of this work in Fig 4 of their earlier study (Goldhill et al. 2018). One way or another, the paper needs to find a way to say more about viruses that people ‘care about,’ even if only indirectly.

**The main aim of this paper was to demonstrate whether a favipiravir resistant virus could transmit successfully between ferrets as a model to understand the probability of favipiravir resistant virus transmitting between humans. We demonstrated that an early H1N1 pandemic strain from 2009 with two mutations can transmit without any obvious fitness cost. In many cases of transmission only the double mutant transmitted to a ferret. Although in ferrets with a mixed population of double mutant and wildtype viruses, there were more complex dynamics which led to an increase in the P653L single mutant, this should not detract from the fact that transmission was possible for this virus. Looking at more recent H1N1s, we show that the P653L mutation is still necessary for full compensation of K229R but did not provide increased polymerase activity on a N321K background. This implies that if favipiravir resistance was generated in a more recent H1N1, it would also transmit easily as we did not see a cost to K229R + P653L in our experiment. Furthermore, it might be less likely to lose the K229R mutation as there is likely no fitness advantage for the P653L single mutant. Our results are therefore directly applicable to currently circulating H1N1 viruses.**

**Goldhill et al. (2018) used H3N2 and the potential pandemic virus H7N9 and found that there was less of a cost to the polymerase of the K229R mutation as was seen in this paper for more a recent H1N1. We have added to our discussion to discuss how favipiravir resistance might arise and transmit in these viruses (L383-393).**

(3) What is the model saying? I am not a specialist in modelling but I found a number of aspects of the model troubling and would like to know the author’s reasoning; I suspect most readers would too.
To begin with, it would be helpful to have a bit more background about the scenarios under which favipiravir resistance would be expected to spread ‘in the wild’ – perhaps by expanding the introductory section on the spread of resistance to previous anti-influenza drugs. Influenza is an acute infection, and favipiravir would be expected to be administered to severely ill patients only in a hospital setting. Even if the drug was used more widely, there are no reasonable circumstances in which the majority of human-to-human influenza transmission events in a human population would occur between favipiravir-treated individuals (in this sense it is very different from evasion of adaptive immunity). There are therefore a limited set of scenarios in which resistance could spread (which, to be clear, is certainly a worrying possibility), for example if the resistant constellation had little or no fitness cost in the absence of treatment, or if prophylactic treatment was excessively used in e.g. susceptible livestock. A brief but clear articulation of the selective pressures under which favipiravir resistance might be expected to spread is key context for the entire paper, but is currently missing.

**The most likely scenario for the development of widespread favipiravir resistance is that resistance develops in a patient treated with favipiravir and then spreads throughout an untreated population. Resistance at the population level could then happen via drift if there is no fitness cost to resistance or selection for a trait associated with the mutations as has been seen in the evolution of resistance to other antivirals. We have added an additional paragraph to the introduction. (L103-114).**

Moving on to the model itself, we have Figure 3, which presents four scenarios all with starting composition 95% (K229R + P653L) to 5% WT:
(a) Relative fitness: P653L (1.25) > WT (1) = K229R+P653L (1) >> K229R (0.01). Reassortants generating K229R (653P) are lost rapidly. Reassortants generating P653L (229K) have a fitness advantage and slowly outcompete the starting WT and K229R+P653L forms. At generation 20 nearly all the population carries 653L, and 229R has declined from 95% to c.60%.
(b) Relative fitness: WT (1) = P653L (1) = K229R+P653L (1) >> K229R (0.01). Reassortants generating K229R (653P) are lost rapidly. Reassortants generating P653L (229K) are in equilibrium with K229R+P653L. WT would be in equilibrium too without reassortment but, due to the large starting amount of K229R + P653L, in any reassortant event WT is likely to mix with a double mutant and so be lost. At generation 20 nearly all the population carries 653L, and 229R remains at 95% but is now largely on a PA 653L background.
(c) Relative fitness: P653L (1.25) > WT (1) = K229R (1) = K229R+P653L (1). Reassortants generating K229R (653P) do not have a fitness disadvantage but are rare events due the starting composition; they behave much as WT in (b). Reassortants generating P653L (229K) have a fitness advantage and exponentially outcompete all other species much as in (a). At generation 20 nearly all the population carries PA 653L (slightly less than in (a)), and PB1 229R, now largely on a PA 653L background has declined from 95% to c.60%.
(d) Relative fitness: P653L (1.25) > WT (1) = K229R+P653L (1) >> K229R (0.01); no reassortment. Here the starting composition of K229R+P653L and WT is in equilibrium, and the single mutants can only be generated by spontaneous mutation at a much lower rate. Where this happens K229R (653P) is rapidly lost, and P653L (229K) once it appears begins to outcompete the other species with similar eventual kinetics as in (a) and (c) but from a much lower starting position. At generation 20 the starting composition is only slightly changed, but if you ran this out to 60 generations the eventual increase of P543L (229K) would presumably end up displacing 229R from the population.
I wouldn’t normally write this out in this much detail in a review, but I want to do so here for three reasons.
First, it took a great deal of work for me to work all of this out (most of an evening), and until I had done so I was entirely unable to make any sense of Figure 3. It should have been evident from the way the paper was written - the explanation needs to be clearer.

**We would like to thank the reviewer for this thorough review of the model. We apologise that the description of the model was not sufficiently detailed. We have significantly expanded our description of the model results (L196-272) as well as our sensitivity analysis (see appendix.)**

Second, note that in the summary above the fitness effects of K229R and P653L are strictly independent. The conclusion that ‘the fitness advantage of the PA P653L single mutant was necessary and sufficient to explain the loss of the R229 PB1 resistance mutation’ (lines 211-3) is not obvious to me. Rather, the models suggest to me that (as is intuitively obvious) K229R is lost because it carries a fitness cost, and that if a large proportion of double mutants are present in the initial mix, they will eventually dominate all reassortment events.

**We apologise that we have not distinguished well enough between the allele K229 and the single mutant K229R. The model was designed to explain the increase in K229 allele in ferrets which was not matched by an increase in the P653 allele. Our model showed that the increase in the K229 allele was caused by an increase in the P653L single mutant. In figure 3b, where there was no fitness advantage to the P653L mutant, we did not see the increase in the K229 allele. Whereas in figure 3c, where we removed the fitness cost for the K229R mutant, the P653 allele was now maintained but there was still an increase in the K229 allele. Therefore, this showed that the increase in the K229 allele was due to the fitness advantage of the P653L mutant. The reviewer is correct that the K229R mutant and the WT virus are selected against due to recombination and the fitness cost of the K229R mutant.**

Third, the outputs of these models are clearly dependent on arbitrary parameters, as any model would be. The authors claim that their sensitivity analysis shows that the model outputs are ‘robust to changes in values of the initial variables’ (line 221). This is simply incorrect (which is actually reassuring, as one would expect a realistic model to be sensitive to these variables). The variable fWM, currently 1.25 (a 1.25-fold increase in fitness), behaves radically differently if increased by just 20% to 1.5. The variable fMW, currently 0.01 (a 100-fold decrease in fitness) is not tested at all, and nor is pMM (the initial proportion of double mutant virus) both of which I would argue have a major effect on the model outcomes. A much more thorough and explicit sensitivity analysis is needed, exploring model outcomes over a range of plausible values. Indeed, this could add to the value of the paper, as it would allow some consideration of what might happen in a favipiravir-treated patient where the relative fitness of WT PB1 will be, by definition, depressed, as well as considering other scenarios (see above).

**We have greatly expanded the sensitivity analysis to examine how changes in reasonable ranges to a large range of parameters affected the model results (see Appendix). We have varied all the input parameters to the model as well as testing different model assumptions. We found that at the extreme ends of the parameter distribution, there were some changes to the model e.g. increasing MOI to 10 resulted in very different dynamics. Increasing *fWM* did not lead to qualitative changes in the baseline model but did affect the final proportions. However, in a model without reassortment, if sufficiently high, *fWM* could cause a large increase in PA P653L. We have now commented on this in the manuscript L307-310.**

**Part III – Minor Issues: Editorial and Data Presentation Modifications**
Please use this section for editorial suggestions as well as relatively minor modifications of existing data that would enhance clarity.

Reviewer #1: 1) In Figure 3, the authors provide a mathematical model of their findings. The model is consistent with the authors results; however, caution needs to be taken when interpreting this model. The model is derived from a single set of parameters in which ferrets were inoculated with a single mixture of viruses at a ratio of 95% mutant viruses to 5% wild-type. The model would benefit from an additional set of data inputs, as this would validate the findings. This is beyond the scope of the manuscript, but the authors should discuss the limitations of using a single set of parameters to develop the model or provide justification for why a single set of parameters is sufficient.

**We have greatly extended the sensitivity analysis for the model including varying the initial inoculum (appendix Figure 4 and 5). We show in appendix Figure 4 that increasing the percentage of WT in the inoculum still results in the loss of WT albeit more slowly. It also leads to an increase in the P653L single mutant as there are more opportunities for early reassortment generating this virus. In the absence of reassortment, increasing the percentage of WT in the inoculum leads to increased WT after 20 generations as WT is not removed by reassortment or efficiently outcompeted.**

Reviewer #2: 1. Line 156 refers to a low number of mutations in recipient ferrets and surmises that their prevalence may be due to transmission bottlenecks. Please elaborate. Were these few mutations present at high frequencies? The prior sentence states that no mutations were found in the pol genes. Were the mutations referred to in other genes? Did their frequency increase over time in the recipient ferrets (evidence of positive selection)?

**There were a few mutations which were present in individual ferrets. These were not in the PB1 and PA segments. The mutations in indirect contact ferrets were almost all fixed at the first time point which suggested that they were as a result of a tight bottleneck and therefore their frequency could not increase over time. The mutations in the direct contact ferrets were polymorphisms indicating a wider bottleneck. There were no mutations found at high frequency in multiple ferrets which could have indicated positive selection. The one exception to this was a mutation found in both the direct and indirect contact for a single donor. Some mutations were present as polymorphisms in direct contact ferrets and were also present in the inoculum e.g. mutation on the NS segment which was present at 1% in the inoculum and transmitted with WT in one contact ferret (353). Without parallel evolution and with only 2 time points, it was not possible to identify positive selection in our sequencing. We have included this information in L165-177.**

2. In the description of the baseline model (ca. line 196), a fitness value is given for only one of the four viruses. Please indicate parameters used for all viruses here. Did the values used correspond quantitatively with the data cited in ref 17? At this location in the text, it would also be helpful include basic information on how MOI was determined and what assumptions were used to govern reassortment rates - or at least refer to the appendix/methods here.

**The fitness values have been added for all the viruses and the reasoning discussed (L219-224). The values used correspond qualitatively to the data from ref 17 as well as the additional data which has now been included in the paper (Supplemental Figure 1). It is difficult to estimate fitness per viral generation in a multigeneration plaque assay and the fitness values for the viruses measured in MDCK cells will not be the same as fitness in a ferret. For this reason, we have used qualitative values rather than directly taking values from a growth curve on MDCK cells. We have also included additional graphs in the sensitivity analysis detailing what happens if these fitness values are varied (see Appendix figures 1-3.)
The MOI was assumed to be relatively high (1) to ensure high levels of reassortment as has been seen from previous studies e.g. Marshall et al. 2013 and Richard et al. 2018. (L211-214, L473-477.)**

3. In the appendix:
A. Please add references indicating the sources of default parameters

**We have added additional references including Jacobs et al. 2019, Pauly et al. 2017, Fukuyama et al. 2015, Handel et al. 2007, Petrie et al. 2013.**

B. Please include results obtained with the default parameters for comparison.

**Default parameters are now included in graphs in the appendix.**

C. The default parameter for viral load seems low. Is the model sensitive to changes in this parameter?

**Figure 8 in the appendix shows that there was no difference when the viral load was varied with reassortment. In the absence of reassortment, there was a very slight increase in the proportion of P653L as mutants will be generated more quickly with a higher viral load. The default parameter was chosen as being a plausible representative of the number of virions that could be found in the respiratory tract as detailed in the appendix.**

D. How would a higher MOI impact results? (MOI may be very high within foci of infection, even in vivo.)

**Interestingly, at a very high MOI of 10 (Appendix Figure 7), our results were qualitatively different. This was because at very high MOI there is little opportunity for selection to take place in our model as the possibility of only a single genotype infecting a cell is unlikely. This dilutes the advantages and costs of different mutations because even if a genotype has a severe fitness cost, it will still be replicated/packaged due to the polymerases from other coinfecting viruses.**

4. This is not absolutely necessary in my opinion, but to really round out the paper, it would be great to see a second (equivalent) ferret experiment done in the Eng687 background to test the prediction that the evolutionary dynamics of the K229R resistance mutation depends on strain background.

**We agree that further ferret experiments would be interesting but unfortunately due to limitations on animal experiments due to the coronavirus pandemic as well as financial and time constraints, we regret that we are unable to perform these experiments at this time.**

5. The discussion nicely discusses the results of the present study but could do more to put these results in context relative to the literature.

**We have added an additional paragraph to the discussion and compared our results to baloxavir resistant virus being transmitted in ferrets as well as oseltamivir resistance in seasonal and pandemic H1N1 viruses. (L375-393).**

Reviewer #3: The arguments in the paper can often be quite hard to follow, something which would be helped considerably by a clear concluding statement at the end of each results section (currently missing from most, though not all).

**We have now added concluding statements to each results section which will hopefully make the argument clearer to follow. ( L160-162, L175-177, L193-194, L238-240, L264-265.)**

The discussion of Figure 3 is currently ‘upside down’ (the text refers to the frequency of K at PB1 229 while the figure shows the frequency of R).
 **We hope that the revamped discussion of Figure 3 is now easier to follow. Figure 3 shows frequency of viruses rather than frequency of the alleles.**

Missing references: Line 100-2: ‘In humans so far…’

**We have now added references here (Ujike et al. 2011 and Uehara et al. 2020).**

Line 302 ‘not being structurally close to the active site’ (in this case a supplementary figure would be ideal).

**Please see supplementary figure 4.**

Line 103-4: ‘equivalent mutation’ please explain
**We have changed the line to explain that the mutation is at a conserved residue in the F-motif L114-117.**

*Interestingly, K229 is a conserved residue in the F-motif of the polymerase(17) and favipiravir resistant chikungunya virus containing the corresponding mutation to K229R in influenza, has been shown to reproduce less efficiently in mosquitos which led to slower transmission**(27).*

Line 140: ‘shortly after infection and a later time point’ – please explain more clearly. It appears to be after the first increase in titre and at the last time point before the final fall in titre, but this isn’t made clear.

**We have clarified which sequences were chosen. The early timepoint was chosen at the first time point with enough viral material for sequencing and the late timepoint was chosen to match the 2^nd^ peak if possible. L150-153.**

Line 143: ‘high levels’; line 156 ‘low number’, line 197 ‘a fitness advantage’ – please quantify (at least approximately)

**I have now quantified these.** **L156, L170-171, L218-224.**

Line 144 -6: ‘indicating that the K229R + P653L virus … was efficiently transmitted…’ given the (reasonable) choice to stack the deck in favour of the mutant virus the fact that it was transmitted does not actually say much about how efficient this was.

**We have changed this sentence to remove discussion of efficiency L155-158.**

Line 156-9: if ‘bottlenecking’ could have removed low-frequency mutations, why is this not also a reasonable explanation for the loss of the WT genome segments?

**This is a reasonable explanation for the loss of WT segments in aerosol transmitted ferrets. It would not explain the changes seen in the donors or contact ferrets.**

Line 159: ‘pattern of repeated mutations’ meaning unclear (to me)

**Mutations weren’t seen in multiple ferrets so it was likely chance and not positive selection which was responsible for any mutations seen. L172-175.**

Line 197 (and elsewhere): I believe that the term ‘fitness’ here should more properly be ‘relative fitness’

**For ease of reading, we have in general left this as fitness. We believe that it is clear from the model that it is relative fitness.**

Why is Figure 5 in the discussion rather than the results and why is only group 1 shown?

**Figure 5 is a schematic using the data from Figure 2 and is an interpretation of the likely viral genotypes based off the modelling from figure 3. Only 1 group is shown as a representative example of the processes discussed in the figure. As did not contain new data but just interpretation of existing data, we thought it was appropriate for the discussion.**

Line 303-5: ‘This might imply… cost.’ Meaning unclear (to me). Due to compensation?

**The cost to polymerase activity of PB1 K229R is less in viruses containing PA K321. Therefore, resistance might evolve more easily as there is less of a cost to developing resistance. L389-393.**

Lines 322-4: ‘Given that… mutations.’ Reasoning unclear (to me).
**Resistance to favipiravir through K229R comes at a cost which is compensated by P653L. As P653L is not present in flu at the moment, it suggests that two mutations would be required to get resistance without a cost (i.e. K229R and P653L). L413-415.**

Line 340: date of download and number of sequences downloaded required
**See below**

Line 341: please briefly explain how the alignment was performed and give the accession number (or other source) of the reference sequence.

**We have now added the following (L431-434.)**

*11,455 pH1N1 (2009) viruses with full length PA segments were downloaded from GISAID on 12/03/2017. They were aligned in Geneious v11 (Map to Reference) to an Eng195 reference (GenBank ID: GQ166654) and all mutants at location 653 were analysed by eye.*

Line 385-6: ‘mean proportional to the summed fitness’ – summed (as opposed to mean) fitness implies that a cell infection with, say, 5 viruses should produce 5 times more progeny than a cell infected with one virus. Is this what was meant? If it is – given that high MOIs of IAV tend in practice to increase the rate of virus production rather than the maximum yield – does this not skew the model excessively to considering reassortant events? (It may be that in practice the nature of the Poisson distribution at MOI 1 means that the vast majority of higher order terms are negligible, but it would be good to know.)

**This is a very fair observation. We have now looked at alternate ways of determining burst size in the appendix (Figure 9) and determined that it did not qualitatively change our results that reassortment is essential.**

Line 393: units for the mutation rate are missing, and there appears to be an additional superscripted term. (See also line 537.)

**Superscripted term was a reference which was accidentally superscripted. There are no units as it was a probability of mutation per generation based off Pauly et al.**

Line 544: how many cells for this amount of DNA?

**These assays were performed in either 24 or 48 well plates giving ~2*10^6 and 1*10^6 cells respectively.**

Figure 4 legend: What is N? What measure of central tendency and variance has been plotted?

**Have added the following to the legend:**

*N = 3. Error bars show s.d. Experiments were repeated 3 times and a representative experiment is shown.*

Line 557-8: Suggest removing interpretation from a figure legend.

**This interpretation has been removed.**

Figure 1: the black outlines are hard to distinguish

**We have now made the outlines thicker.**

Appendix: Keys are missing for the colours in the figures.

**Keys have been added to all the figures in the appendix.**
